# Lifelong versus not lifelong death wishes in older adults without severe illness: a cross-sectional survey

**DOI:** 10.1186/s12877-022-03592-5

**Published:** 2022-11-21

**Authors:** Elke Elzinga, Margot Zomers, Kiki van der Burg, Sisco van Veen, Lizanne Schweren, Ghislaine van Thiel, Els van Wijngaarden

**Affiliations:** 1 Research Department, 113 Suicide prevention, Paasheuvelweg 25, 1105 BP Amsterdam, the Netherlands; 2 Department of Psychiatry, Amsterdam University Medical Center, Vrije Universiteit Amsterdam, Amsterdam Public Health Research Institute, De Boelelaan 1117, 1081 HV Amsterdam, the Netherlands; 3 Department of Public Health, Healthcare Innovation & Evaluation and Medical Humanities, Julius Center for Health Sciences and Primary Care, University Medical Center Utrecht, Universiteitsweg 100, 3584 CX Utrecht, the Netherlands; 4 Department of Psychiatry, University Medical Center Utrecht, Heidelberglaan 100, 3584 CX Utrecht, the Netherlands; 5 Department of Psychiatry & Department of Ethics, Law and Humanities, Amsterdam University Medical Center, De Boelelaan 1117, 1081 HV Amsterdam, the Netherlands; 6 Department of Anesthesiology, Pain and Palliative Medicine, Radboud University Medical Center, Geert Grooteplein Zuid 10, 6525 GA Nijmegen, the Netherlands

**Keywords:** Death wish, Suicide ideation, End-of-life, Old, Lifelong, Long-lasting

## Abstract

**Background:**

Some older adults with a persistent death wish without being severely ill report having had a death wish their whole lives (lifelong persistent death wish; L-PDW). Differentiating them from older adults without severe illness who developed a death wish later in life (persistent death wish, not lifelong; NL-PDW) can be relevant for the provision of adequate help and support. This study aims to gain insight into the characteristics, experiences, and needs of older adults with a L-PDW versus older adults with a NL-PDW and into the nature of their death wishes.

**Methods:**

In the Netherlands, in April 2019, a cross-sectional survey study was conducted among a large representative sample of 32,477 citizens aged 55 years and older. Respondents with a L-PDW (*N* = 50) were compared with respondents with a NL-PDW (*N* = 217) using descriptive statistics, Kruskal–Wallis tests, and Fisher’s exact tests.

**Results:**

Respondents with a L-PDW were relatively younger and less often had (step)children. They less often looked back on a good and satisfying life with many good memories and more often reported trauma. Older adults with a NL-PDW more often reported loss and bereavement. Overall, the groups showed a lot of similarities. Both groups reported a death wish diverse in nature, numerous health problems, and a variety of needs for help and support.

**Conclusions:**

Some of the differences we found between the groups might be particularly relevant for the provision of adequate help and support to older adults with a L-PDW (i.e., their past and trauma) and to older adults with a NL-PDW (i.e., their loss and bereavement). The heterogeneity of both groups and the diverse nature of their death wish indicate that careful assessment of the death wish, its background, and underlying needs is required to provide personalized help and support to older adults with a death wish.

**Supplementary Information:**

The online version contains supplementary material available at 10.1186/s12877-022-03592-5.

## Background

Death wishes occur regularly among older adults. Pooled results from 11 population studies across Europe (15.890 respondents) showed that 6% of adults aged 65 and above reported a death wish. This rate ranged from 3 to 27% across different countries [1]. In the Netherlands, 4% of the adults aged between 57 and 99 years old reported a current wish to die or a weakened wish to live and 12% reported having experienced death thoughts or a death wish at some point in their lives [2].

A death wish can be described as a longing for death for oneself. If someone expresses a death wish this can have various reasons, meanings, functions, and underlying intentions [3, 4]. Death wishes can be both passive and active. Passive death wishes may range from the belief that life is not worth living to thoughts of or desire for death. Death wishes are deemed *active* if people have made concrete plans or taken steps regarding their death wish and/or have seriously considered attempting suicide [5, 6]. However, a universal and widely acknowledged definition of death wishes and suicide ideation and the distinction between both is lacking [5, 7, 8].

Death wishes at an older age can originate from several reasons. Research among older adults showed that death wishes and suicidal feelings are, among others, associated with physical and mental illness [2, 9–12]. However, death wishes also occur among older adults who are not (severely) ill [2]. The terms “completed life” or “tiredness of life” are often used to describe death wishes of older adults whose suffering is not predominantly caused by medically classifiable conditions [13, 14].

In the Netherlands, a cross-sectional survey study was conducted among a large representative sample of 32,477 citizens to gain insight into adults aged 55 years and older who developed a persistent death wish (≥ 1 year) while not being severely ill (PDW-NSI). This study showed that approximately 1% of the older adults had a PDW-NSI. The authors also detected a subgroup of older adults whose death wish was not specifically related to old age, since almost one in five respondents with PDW-NSI reported having had a death wish their whole lives. To our knowledge, this study was the first to describe a relatively large group of older adults with a lifelong persistent death wish (L-PDW) [6].

Death wishes often represent various ways of suffering and distress [15–18]. Experiencing a death wish may increase the likelihood of engaging in suicidal behaviour, especially when present from an early age [19–22]. This illustrates the importance of identifying and supporting people with a death wish.

Differentiating older adults with a L-PDW from older adults who have a persistent death wish, not lifelong (NL-PDW), can be relevant for the provision of adequate help and support. We, therefore, performed a secondary explorative analysis of data that was gathered by Hartog et al. [6] in which we compared older adults with a L-PDW with older adults with a NL-PDW. The aim of this study is to gain insight into the characteristics, experiences, and needs of older adults with a L-PDW versus older adults with a NL-PDW and into the nature of their death wishes.

## Methods

### Study design and population

A group of people aged 55 years and older (*N* = 32,477), representative for the Dutch population of older adults, were approached via research company Kantar Public to complete a questionnaire (additional fig. 1) [23]. Between April 3 and April 25, 2019, 21,294 respondents (65.6%) completed this questionnaire.

### Questionnaire

The questionnaire included items about background characteristics, health and illness, the nature of the death wish, needs for help and support, aspects strengthening the wish to die or to live, perspective on life, good memories, negative experiences or events, and life goals. For health and illness, several validated health indicators were included: the Visual Analogue Scale (VAS) to measure the current health state of the respondents with scores ranging from 0 (worst imaginable health state) to 10 (best imaginable health state). The EuroQol EQ-5D-5L was used to measure the health state on five different domains: mobility, selfcare, daily activities, pain, and mood. Sum scores range from 5 to 25 and higher scores indicate more severe problems ﻿[24]. The Depression subscale of the Hospital Anxiety and Depression Scale (HADS), which was used to assess the probability of a depression, ranges from 0 to 21. Higher sum scores are related to a possible indication for a severe depression [25]. Respondents were asked whether they (had) suffered from a life-threatening disease. Further, they were asked to select the diseases, complaints, and medications that applied to them from a list and to report the burden of these diseases and complaints using a 10-point scale ranging from 1 (“very little”) to 10 (“very much”).

The nature of the death wish was captured by assessing the duration, respondents’ characterization of the death wish, the frequency of thinking about it, and the (relative) strength and alternation of the death wish and wish to live. Furthermore, respondents were asked whether they had made concrete plans or taken steps concerning their death wishes, had considered or attempted suicide in the past year, and had communicated about their death wishes.

Needs for help and support were assessed and compared by means of a list of response options in which respondents could select multiple needs. The same holds for many items displayed in the tables in the addendum: respondents could select multiple answers from a list to indicate their diseases, complaints, and medications; aspects strengthening their wishes to die or to live; good memories; and negative experiences or events. To describe their perspectives on life and life goals, respondents indicated the extent to which an answer applied to them by means of 7-point Likert scales.

For a full description of the questionnaire, see van Wijngaarden et al. [26].

### Participants

The questionnaire included a differentiation question to select the group of interest from the total group of respondents: *“Does the description ‘seeing no future for oneself, longing for death, while not being severely ill’ apply to you at this moment?”* This differentiation question was based on the descriptions of “completed life” and “tiredness of life” as used in literature [13, 14]. Having a death wish was operationalized as a “longing for death” for oneself [6].

After selecting respondents who answered the differentiation question affirmatively (additional fig. 1), three additional inclusion criteria were applied (additional Fig. 2). Respondents who 1) reported no severe health problems (VAS score ≥ 4 and EQ-5D sum score < 17), 2) showed no indication for severe depression (HADS depression subscale sum score < 16), and 3) reported having a persistent death wish for 1 year or longer (PDW) were selected. This resulted in the group of interest of *N* = 267 (1.25% of the total response). For the current analysis, these respondents were divided into two groups. Respondents who selected “Basically my entire life” as answer to the question “How long have you had a wish to be dead?”, were classified as the lifelong (L)-PDW group; respondents who selected either “For several years” or “About one year”, were classified as respondents with a persistent death wish, not lifelong (NL)-PDW.

### Data analysis

In this paper we present descriptive statistics, including frequencies (percentages) and medians (with interquartile range). The two groups were compared using either Kruskal–Wallis tests for ordinal variables or Fisher’s exact tests for nominal variables. Since this is an explorative study for which we did not formulate hypotheses, we did not apply statistical corrections for multiple comparisons. Statistical significance was determined using 0.05-level two-sided tests. All analyses were performed using SPSS software, version 26.0.0.1.

For a more extensive description of the methodology of the primary study, see Hartog et al. [6].

## Results

Respondents with a lifelong persistent death wish (L-PDW: *N* = 50) were compared with respondents with a persistent death wish, not lifelong (NL-PDW: *N* = 217). In the NL-PDW group, *N* = 49 (23%) indicated having had a death wish for about one year and *N* = 168 (77%) for several years.

Table 1 shows the background characteristics of all respondents. Respondents in the L-PDW group were significantly younger: their median age was 62 years compared with 67 years for respondents with a NL-PDW (*P* < 0.001 Kruskal–Wallis test). In addition, they significantly less often had (step)children (38% vs 17% had no (step)children, *P* = 0.001 Fisher’s exact test).

**Table 1:** Background characteristics

	**L-PDW (*N* = 50)** N (%)	**NL-PDW (*N* = 217)** N (%)	***P*-value**
Gender			
Female	33 (66)	116 (53)	0.117 (F)
Male	17 (34)	101 (47)	
Age (years)			
Median (Q1-Q3)	62 (58–68)	67 (61–75)	**0.000** (K)
55–59	19 (38)	39 (18)	
60–64	14 (28)	52 (24)	
65–69	7 (14)	39 (18)	
70–74	9 (18)	32 (15)	
75–79	0 (0)	32 (15)	
80 +	1 (2)	23 (11)	
Educational attainment^a^			
Low	16 (32)	82 (38)	0.108 (K)
Middle	14 (28)	83 (38)	
High	19 (38)	50 (23)	
Worldview^b^			
Religious worldview	21 (40)	85 (37)	0.750 (F)
Non-religious worldview	15 (29)	66 (29)	1.000 (F)
No worldview	15 (29)	70 (30)	0.867 (F)
Worldview, religiousness unknown	1 (2)	10 (4)	0.695 (F)
(Step)Children^c^			
No	19 (38)	37 (17)	**0.001** (F)
Yes	19 (38)	131 (60)	
Household size			
Living alone	29 (58)	110 (51)	0.433 (F)
Living not alone	21 (42)	107 (49)	
Social class^d^			
Low	24 (48)	118 (54)	0.278 (K)
Middle	8 (16)	30 (14)	
High	18 (36)	69 (32)	
Urbanization^e^			
Very high	19 (38)	58 (27)	0.180 (K)
High	13 (26)	70 (32)	
Moderate	9 (18)	36 (17)	
Low/none	9 (18)	53 (24)	

Results are presented as N (%) unless “Median (Q1-Q3)” is reported

Percentages may not add up to 100% because of rounding

Medians are reported with 25th-75th percentiles

Statistically significant results (*p* < 0.05) are in bold. *P*-values determined by Fisher’s exact tests are indicated with (F) and P-values determined by Kruskal–Wallis tests with (K)

^a^ Low = lower vocational education, lower secondary education, or lower. Middle = intermediate vocational education or higher secondary education. High = higher vocational education or university. *N* = 1 and *N* = 2 respectively selected “I do not know/want to answer”. This category was not included in the test

^b^ Religious worldview = Protestant, Catholic, Muslim, Jewish, Hindu and Buddhist. Non-religious worldview = atheist, agnostic, “spiritual but not religious”, humanist, anthroposophical and esoteric. Worldview, religiousness unknown = other worldview. Respondents could give more than one answer and may thus be counted in more than one category. Therefore, *N* = 52 and *N* = 231 respectively and percentages are based on these numbers. In group comparisons, worldview is tested with separate tests for each category (yes/no)

^c^ *N* = 12 and *N* = 49 respectively are missing

^d^ Based upon the educational attainment and profession of the main breadwinner in the household

^e^ Very high= $>$ 2500, high = 1500–2500, moderate = 1000–1500, low/none =  < 1000 addresses per km^2^

Table 2 provides information about the health and illness of respondents in the L-PDW and NL-PDW groups. None of these health and illness parameters differed significantly between groups. Additional table 1 describes the occurrence of certain diseases and complaints in these groups and their use of certain medications. This table shows, for example, that hearing and vision problems were significantly more often reported by respondents with a NL-PDW compared with those with a L-PDW (*P* = 0.003 and *P* = 0.025, respectively Fisher’s exact tests).

**Table 2:** Health and illness

	**L-PDW (N = 50)** N (%)	**NL-PDW (*N* = 217)** N (%)	***P*-value**
Current health state VAS			
Median (Q1-Q3)	7 (6–7)	6 (5–7)	0.512 (K)
EQ-5D-5L sum score			
Median (Q1-Q3)	10 (8–12)	10 (8–13)	0.505 (K)
HADS depression subscale, sum score			
Median (Q1-Q3)	10 (4–12)	10 (7–12)	0.204 (K)
Life-threatening disease			
Never	40 (80)	159 (73)	0.472 (F)
Yes, but not anymore	9 (18)	43 (20)	
Yes, at this moment	1 (2)	15 (7)	
Number of current diseases^a^			
None	4 (8)	21 (10)	1.000 (F)
Median (Q1-Q3)	2 (1–3)	2 (1–3)	0.810 (K)
Burden of current diseases			
Median (Q1-Q3)	7 (5–8)	7 (5–8)	0.827 (K)
Number of current complaints^a^			
None	2 (4)	6 (3)	0.646 (F)
Median (Q1-Q3)	4 (2–6)	5 (3–7)	0.135 (K)
Burden of current complaints			
Median (Q1-Q3)	7 (5–7)	6 (5–7)	0.493 (K)
Number of medications^a^			
None	15 (30)	39 (18)	0.077 (F)
Median (Q1-Q3)	3 (2–4)	2 (1–4)	0.448 (K)

Results are presented as N (%) unless “Median (Q1-Q3)” is reported

Percentages may not add up to 100% because of rounding

Medians are reported with 25th-75th percentiles

Statistically significant results (*p* < 0.05) are in bold. P-values determined by Fisher’s exact tests are indicated with (F) and *P*-values determined by Kruskal–Wallis tests with (K)

^a^ Listed in additional table 1

Results regarding the nature of the death wish are presented in Table 3. The characterization of the death wish differed significantly between the two groups (*P* = 0.025 Fisher’s exact test). In addition, respondents with a NL-PDW indicated a significantly stronger death wish in the past week (*P* = 0.046 Kruskal–Wallis test). The groups did not differ significantly in terms of having made concrete plans or taken steps concerning the death wish, or in terms of having seriously considered attempting suicide. Of the respondents who ever considered attempting suicide, three (all from the NL-PDW group) attempted suicide in the past 12 months. With regard to communication about the death wish, the L-PDW group had significantly more often discussed the death wish than the NL-PDW group (24% vs 42% respectively had not discussed the death wish, *P* = 0.024 Fisher’s exact test).

**Table 3:** Nature of the death wish

	**L-PDW (*N* = 50)** N (%)	**NL-PDW (*N* = 217)** N (%)	***P*-value**
Characterization of the death wish			
A desire for a natural death that just happens	13 (26)	37 (17)	**0.025**
A desire to not wake up tomorrow and die in my sleep	20 (40)	118 (54)	
I feel my current situation is unliveable	1 (2)	10 (5)	
A wish to end my life myself	10 (20)	19 (9)	
A wish for someone to help me end my life^a^	1 (2)	21 (10)	
I do not know	5 (10)	12 (6)	
Frequency of thinking about the death wish			
Rarely	11 (22)	19 (9)	0.111
Every month	5 (10)	21 (10)	
Every week	12 (24)	46 (21)	
Every day	3 (6)	31 (14)	
All the time	1 (2)	5 (2)	
It varies, sometimes frequently, sometimes not very often	18 (36)	95 (44)	
Strength of the death wish in the past week			
Median (Q1-Q3)	5 (2–6)	5 (3–7)	**0.046**
Strength of the wish to live in the past week			
Median (Q1-Q3)	5 (3–7)	5 (3–6)	0.938
Relative strength of the death wish and the wish to live in the past week			
The wish to live was stronger	21 (42)	74 (34)	0.467
About the same	19 (38)	102 (47)	
The death wish was stronger	10 (20)	41 (19)	
Alternation between the death wish and the wish to live			
In my case the death wish is always stronger	7 (14)	40 (18)	0.572
In my case the wish to live is always stronger	7 (14)	21 (10)	
Some periods my wish to live is stronger, at other times my death wish is dominant	36 (72)	156 (72)	
Having made concrete plans/taken steps concerning the death wish			
No	42 (84)	174 (80)	0.690
Yes	8 (16)	43 (20)	
Having seriously considered attempting suicide in the past 12 months^b^			
Never	23 (46)	90 (42)	0.749
Ever	25 (50)	110 (51)	
Having made a suicide attempt in the past 12 months^c^			
Yes	0 (0)	3 (1)	0.507
No	25 (50)	100 (46)	
I don’t want to answer this question	0 (0)	7 (3)	
Communication about the death wish			
No, I have not discussed it with anyone	12 (24)	91 (42)	**0.024**
Yes, with my spouse/partner	15 (30)	42 (19)	0.124
Yes, with (a) sibling(s)	4 (8)	18 (8)	1.000
Yes, with (a) friend(s)	13 (26)	34 (16)	0.099
Yes, with my child(ren) and grandchild(ren)	2 (4)	34 (16)	**0.036**
Yes, with my doctor and/or other health care professionals	11 (22)	66 (30)	0.299
Other	6 (12)	6 (3)	**0.012**

Results are presented as N (%) unless “Median (Q1-Q3)” is reported

Percentages may not add up to 100% because of rounding. Percentages reported for the variable “Communication about the death wish” add up to more than 100% because respondents could select multiple ways of communication

Medians are reported with 25th-75th percentiles

Statistically significant results (*p* < 0.05) are in bold. All were determined by Fisher’s exact tests, except for the two variables “Strength of the death wish in the past week” and “Strength of the wish to live in the past week” (Kruskal–Wallis tests)

^a^ Someone may include a doctor, another professional or someone close

^b^ *N* = 2 and *N* = 17 respectively selected “Not willing to answer”

^c^ *N* = 0 and *N* = 7 respectively selected “Not willing to answer”. Only the respondents who selected “Ever” on the item “Having seriously considered attempting suicide in the past 12 months” (*N* = 25 and *N* = 110 respectively) were asked whether they had actually attempted suicide in the past 12 months

Table 4 presents the needs for help and support that were indicated by the respondents. The groups did not differ significantly in terms of their needs: both groups most often reported a need to have access to a suicide drug, followed by acknowledgement and appreciation of their feelings, assistance from a doctor to commit suicide, and more financial leeway.

**Table 4:** Needs for help and support

	**L-PDW (*N* = 50)** N (%)	**NL-PDW (*N* = 217)** N (%)	***P*-value**
Needs concerning ending one’s life			
Assistance from a doctor to commit suicide	8 (16)	60 (28)	0.106
Assistance from another professional or someone close to you to commit suicide	2 (4)	28 (13)	0.084
Access to a suicide drug	27 (54)	98 (45)	0.275
Information or support to stop eating and drinking	0 (0)	14 (7)	0.079
Social needs			
More social contacts	9 (18)	35 (16)	0.833
Better/more contact with my children/grandchildren/relatives	8 (16)	28 (13)	0.646
Acknowledgement and appreciation of my feelings	14 (28)	50 (23)	0.466
Good conversations with other elderly people/others in the same situation	7 (14)	28 (13)	0.818
Good conversations with a professional (e.g., GP, psychologist or spiritual counsellor)	8 (16)	41 (19)	0.839
Needs for activities			
Meaningful activities	4 (8)	27 (12)	0.469
Meaningful volunteer work	4 (8)	11 (5)	0.492
Opportunities to carry out my hobbies	3 (6)	11 (5)	0.730
Needs for care and guidance			
Meditation or mindfulness training	4 (8)	14 (7)	0.754
More/better professional care and support (e.g., mental or physical)	7 (14)	28 (13)	0.818
Better fine-tuning of medications	4 (8)	28 (13)	0.470
Needs for practical or material things			
More financial leeway	11 (22)	40 (18)	0.554
Another place to live	4 (8)	18 (8)	1.000
Better access to transportation (e.g., public transportation, regional taxi, senior transportation service)	4 (8)	11 (5)	0.492
Other			
Other than above^a^	3 (6)	9 (4)	0.703
I don’t know	3 (6)	10 (5)	0.715
No need for support or assistance	5 (10)	25 (12)	1.000

Results are presented as N (%)

Percentages add up to more than 100% because respondents could select multiple needs

Statistically significant results (*p* < 0.05) are in bold. All were determined by Fisher’s exact tests

^a^ Including being left alone, support in housekeeping, finding a life partner, overcome fears, and to be released of the pressure of providing informal care

Additional table 2 shows aspects strengthening the wish to die or to live. The NL-PDW group significantly more often chose “Loss of my loved ones (e.g., through death, divorce)” as an aspect strengthening their wishes to die and “Good memories (e.g., of the past)” as an aspect strengthening their wishes to live. The L-PDW group significantly more often chose “Sense that I am part of a larger whole” as an aspect strengthening their wishes to live.

Respondents’ perspective on life, good memories, negative experiences or events, and life goals are presented in additional tables 3,4,5,6. Concerning perspective on life, respondents with a NL-PDW significantly more strongly indicated having had a good life, being satisfied with their lives, having good memories, worrying about their partners’ or (grand)children’s’ future, preferring not to have to experience the future, and becoming increasingly dependent on others. Those with a L-PDW on the other hand significantly more strongly indicated being able to take care of themselves. As negative experiences or events, respondents in the L-PDW group significantly more often indicated a trauma and respondents in the NL-PDW group loss or bereavement.

## Discussion

About one-fifth of the older adults with a persistent death wish without being severely ill, reported having had a death wish their whole lives. This study aimed to explore potential differences between older adults with a lifelong persistent death wish (L-PDW) and older adults who developed a persistent death wish later in life (NL-PDW). In general, the groups were largely similar. Both groups were heterogeneous and the nature of their death wishes was diverse. Some differences were found with regard to background characteristics, the nature of the death wish, physical complaints, aspects strengthening the wish to die or to live, perspective on life, and negative experiences or events.

Those with a L-PDW were relatively younger older adults compared with those with a NL-PDW. The fact that they more often reported being able to take care of themselves and less frequently mentioned loss or bereavement as negative experience or event may be associated with this age difference. The age difference may be caused by more suicide deaths in the L-PDW group. After all, especially when the death wish developed at a young age, desire for death is an important risk factor for suicide attempts [22]. However, no one in the L-PDW group attempted suicide in the past year, yet three older adults with a NL-PDW did. This may indicate more active death wishes in the NL-PDW group, though this was not represented in a significant difference. There were also no significant differences between both groups in terms of having made other concrete plans or having taken steps concerning the death wish and having seriously considered attempting suicide in the past year. Altogether, these findings suggest no difference between both groups in how active their death wishes are. In both groups the majority had not made concrete plans or taken steps concerning the death wish and approximately half had not seriously considered suicide in the past year, which points towards the existence of both passive and active death wishes in the two groups. Our results further indicate that a longer duration of the death wish does not necessarily result in a stronger death wish. In fact, the NL-PDW group reported a stronger death wish in the past week and a stronger preference not to have to experience the future.

Older adults with a L-PDW less often looked back on a good and satisfying life with many good memories than older adults with a NL-PDW. They more often reported trauma. Previous research describes links between trauma and adverse childhood experiences, developing a death wish, chronic suicidal thinking, a depressed affect, and mental disorders such as mood and anxiety disorders, posttraumatic stress disorder, and substance use disorders [27–32]. In an interview study of Rurup et al. about wishes to die among older people, of the 31 included interviewees, four reported serious thoughts about death from a very early age. These four respondents related their death wish to traumatic experiences that happened at early age, such as (sexual) abuse or being imprisoned in a Japanese concentration camp. They reported that their death wish remained present throughout their lives, sometimes in the background, but could become more pronounced after experiencing negative life events, like the death of their spouses [31].

Despite these findings of a more troubled past and trauma among older adults with a L-PDW and the specific mental disorders associated with those [32, 33], we did not find differences in terms of mental illness and associated medications between the groups L-PDW and NL-PDW. Approximately half of both groups reported mood or anxiety problems and depression or depressive feelings, and their HADS depression subscale sum scores were similar. Although just above the cutoff point for statistical significance, the L-PDW group more often used antidepressants (43%) than the NL-PDW group (29%), which may indicate differences in their mental health states.

The fact that we also found mood or anxiety problems and depression or depressive feelings among older adults with a NL-PDW may be related to their reports of loss and bereavement. Half of the older adults with a NL-PDW reported “Loss of my loved ones (e.g., through death, divorce)” as an aspect strengthening their wishes to die and they more often indicated loss or bereavement as negative experience or event than older adults with a L-PDW. Bereavement is related to various psychological reactions, such as anxiety and depressive symptoms, and to an increased mortality risk from diverse causes, including suicide. While most people recover from loss over time, for others bereavement can be long-lasting and recovery may take months or even years [34]. Death or divorce of one’s partner can be a trigger for developing a death wish, for example, because life can be experienced as not worth living without one’s partner [31].

## Strengths and limitations with recommendations for future research

To our knowledge, this study is the first to describe many aspects of a L-PDW among older adults without severe illness. It provides explorative insight into the similarities and differences between older adults with a L-PDW and older adults with a NL-PDW. Future research into death wishes can build on this knowledge.

In this study, we focused on differences that were *statistically* significant. This does not necessarily mean that any of the other results which did not reach statistical significance do not differ in real world samples of older adults with L-PDW and NL-PDW. Despite the initial large total group of respondents (*N* = 21.294), the group of 50 respondents with a L-PDW can be considered small for statistical analyses. This may have led to underreporting of differences; i.e., real-world differences that did not appear in this study. Second, differentiating L-PDW from NL-PDW was based upon respondents’ self-reported response to the item “How long have you had a wish to be dead?”. The response was not validated with an open answer option, so it was impossible to verify whether “lifelong” was in fact lifelong, or to provide a more meaningful estimate of the length of the respondents’ death wish. Further research is needed to build on the explorative insight our study offers. Besides more larger-sampled quantitative studies, longitudinal methods and qualitative studies are advised to gain in-depth understanding of the experiences of people with lifelong death wishes, more knowledge of how these death wishes progressed during their lifetimes, and insight into associated risk factors.

Another limitation of this study concerns the findings regarding mental health. Respondents with an indication for severe depression were excluded from the study, which may have diminished differences in mental health factors between the groups. One of the groups may contain more respondents with a severe depression, however, since they were excluded, our study fails to demonstrate this. Second, besides mood or anxiety problems and depression or depressive feelings, no other mental disorders or complaints were assessed. It could be that one of the groups contains more respondents with mental illness we did not measure. Future studies are recommended to include more mental health parameters; this can yield important insights for the provision of adequate help and support to older adults with both L-PDW and NL-PDW.

## Implications for (clinical) practice

Our study offers several relevant insights for (clinical) practice. First, the fact that even though the majority of both groups discussed their death wishes, only a small proportion shared it with a health care professional. Stigma is a commonly reported barrier for disclosing suicide related thoughts. A recent qualitative study described how participants experienced stigma in response to disclosing such thoughts. They, for instance, lost their jobs or the support of friends and family members. Health care was also a source of stigma where prejudice and discrimination occurred, including experiences of participants with health care professionals who did not want to treat them after disclosing previous suicidality [35].

Second, while only a small proportion of both groups discussed their death wishes with a health care professional, the large majority in both groups reported needs for help and support, among others, from health care professionals. Because studies have shown that patients are unlikely to disclose suicidal ideation to their health care providers unsolicitedly [36], health care professionals are advised to actively inquire about death wishes among older adults. Besides facilitating identification of older adults with a death wish, this can also serve as a first step towards an open conversation about the death wish and an exploration of its background and underlying needs. This may enable a personalized approach to help and support, which is recommended given the heterogeneity of both groups and the diverse nature of their death wishes.

Third, our study raises the question whether particularly older adults with a L-PDW may, in some cases, benefit from help and support directed towards coping with their past and trauma and whether particularly older adults with a NL-PDW may, in some cases, benefit from help and support directed towards coping with their loss and bereavement. Furthermore, in addition to the mental health problems described above, both groups reported physical health problems, such as joint problems, neck or back problems, overweight or obesity, chronic pain, and sleeping problems. Regarding physical health problems, the NL-PDW group more often reported hearing and vision problems than the L-PDW group, which may be related to their relatively older age. Providing help and support to, where possible, relieve (age-related) physical and mental health problems, may potentially also positively influences their death wishes. Specifically, because approximately half of both groups indicated diseases and physical or mental deterioration as aspects strengthening the wish to die.

Lastly, almost nine out of ten respondents from both groups reported at least one need for help and support. They not only reported needs concerning the help and support of health care professionals. For example, the needs for acknowledgement and appreciation of their feelings and more financial leeway suggest that besides the formal care network, the social environment and society at large can play a role in meeting the needs of older adults with a death wish. The social environment of these older adults may be small though, since they frequently mentioned the loss of loved ones, not having enough good social contacts, and not having (step)children. Besides, also here, communication barriers may exist, as part of the older adults in our study did not communicate about their death wishes with anyone and indicated to feel like being a burden to others.

## Conclusions

In conclusion, our study shows some differences between older adults with a lifelong persistent death wish and older adults who developed a persistent death wish later in life, with potential opportunities for providing them with adequate help and support. Overall the two groups are similar in terms of reporting a variety of characteristics, experiences, and needs. The heterogeneity of both groups and the diverse nature of their death wishes indicate that careful assessment of the death wish, its background, and underlying needs is required to provide personalized help and support. Besides the formal care network, the social environment and society at large can play a role in meeting the needs of older adults with a death wish, perhaps relieving their death wish.

## Supplementary Information

**Additional file 1:** **Figure 1.** Flowchart sample and response.

**Additional file 2:** **Figure 2.** Flowchart selection process.

**Additional file 3:** **Table1.** Diseases, complaints, and medications.

**Additional file 4:** **Table2.** Aspects strengthening the wish to die or to live.

**Additional file 5:** **Table3.** Perspective on life.

**Additional file 6:** **Table 4.** Good memories.

**Additional file 7:** **Table5.** Negative experiences or events.

**Additional file 8:** **Table 6.** Life goals.

## Data Availability

The datasets generated during and/or analysed during the current study are not publicly available due to the fact that the project analyses have not been completed but are available from the corresponding author on reasonable request.

## Abbreviations

PDW-NSI: Group of persons with a persistent death wish and no severe illness
L-PDW: Subgroup of persons with a lifelong persistent death wish
NL-PDW: Subgroup of persons with a persistent death wish, not lifelong
VAS: Visual Analogue Scale
HADS: Hospital Anxiety and Depression Scale
