# Supplementary figures and images for: Lifelong versus not lifelong death wishes in older adults without severe illness: a cross-sectional survey
Source: BMC Geriatr. 2022 Nov 21;22:885. doi: 10.1186/s12877-022-03592-5 (PMC9680128; doi:10.1186/s12877-022-03592-5)

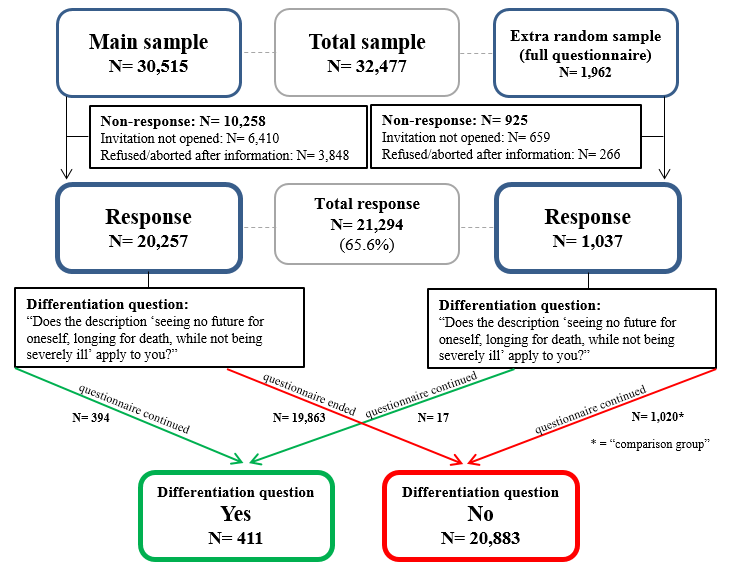


Figure 1 - Flowchart sample and response

Supplement: Supplementary file 1 — Additional file 1: Figure 1. Flowchart sample and response. [file 12877_2022_3592_MOESM1_ESM.docx]

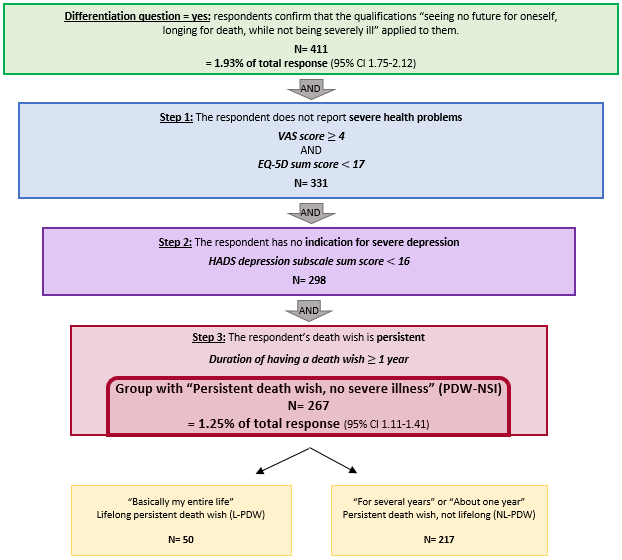


Figure 2 - Flowchart selection process

Total response: N= 21,294.

Supplement: Supplementary file 2 — Additional file 2: Figure 2. Flowchart selection process. [file 12877_2022_3592_MOESM2_ESM.docx]
