# Supplementary material for: Lifelong versus not lifelong death wishes in older adults without severe illness: a cross-sectional survey
Source: BMC Geriatr. 2022 Nov 21;22:885. doi: 10.1186/s12877-022-03592-5 (PMC9680128; doi:10.1186/s12877-022-03592-5)
Supplement: Supplementary file 3 — Additional file 3: Table1. Diseases, complaints, and medications. [file 12877_2022_3592_MOESM3_ESM.docx]

Additional table 1. Diseases, complaints, and medications

|  | | **L-PDW**  **(N=50)** N (%) | **NL-PDW (N=217)**  N (%) | | **P-value** | |
| --- | --- | --- | --- | --- | --- | --- |
| **Diseases** | |  |  | |  | |
|  | **Joint problems (e.g., arthritis, gout, rheumatism)** | 18 (36) | 92 (42) | | 0.430 | |
|  | **Neck or back problems** | 20 (40) | 90 (42) | | 0.875 | |
|  | **Bone decalcification (e.g., osteoporosis)** | 2 (4) | 27 (12) | | 0.127 | |
|  | **Diabetes** | 8 (16) | 33 (15) | | 0.831 | |
|  | **Tightness of the chest (e.g., COPD, asthma)** | 9 (18) | 37 (17) | | 0.838 | |
|  | **Crohn’s disease** | 3 (6) | 3 (1) | | 0.082 | |
|  | **MS/ALS** | 1 (2) | 3 (1) | | 0.566 | |
|  | **Skin disease** | 4 (8) | 11 (5) | | 0.492 | |
|  | **Thyroid problems** | 5 (10) | 22 (10) | | 1.000 | |
|  | **Heart failure/heart disease** | 4 (8) | 31 (14) | | 0.351 | |
|  | **Consequences of a cerebral infarction / brain haemorrhage** | 2 (4) | 11 (5) | | 1.000 | |
|  | **Dementia** | 2 (4) | 6 (3) | | 0.646 | |
|  | **Parkinson’s disease** | 1 (2) | 2 (1) | | 0.465 | |
|  | **Cancer** | 1 (2) | 11 (5) | | 0.475 | |
|  | **Psychological complaints (mood or anxiety problems, depression)** | 27 (54) | 91 (42) | | 0.155 | |
|  | **Other** | 9 (18) | 29 (13) | | 0.377 | |
|  | **None of these** | 4 (8) | 21 (10) | | 1.000 | |
| **Complaints** | |  | |  | |  |
|  | **Hearing problems or deafness, tinnitus** | 8 (16) | 83 (38) | | **0.003** | |
|  | **Eye problems and visual impairment** | 8 (16) | 69 (32) | | **0.025** | |
|  | **Memory problems** | 10 (20) | 67 (31) | | 0.166 | |
|  | **Difficulty speaking** | 2 (4) | 5 (2) | | 0.619 | |
|  | **Headache** | 13 (26) | 53 (24) | | 0.856 | |
|  | **Sleep problems** | 23 (46) | 104 (48) | | 0.876 | |
|  | **Falls (or fear of falling)** | 4 (8) | 43 (20) | | 0.062 | |
|  | **Problems walking** | 18 (36) | 83 (38) | | 0.872 | |
|  | **Dizziness** | 15 (30) | 47 (22) | | 0.264 | |
|  | **Problems with particular movements** | 13 (26) | 77 (36) | | 0.246 | |
|  | **Depression (depressive feelings)** | 28 (56) | 125 (58) | | 0.875 | |
|  | **Lack of appetite** | 5 (10) | 26 (12) | | 0.810 | |
|  | **Overweight, obesity** | 15 (30) | 65 (30) | | 1.000 | |
|  | **Incontinence (urinary or bowel)** | 4 (8) | 19 (9) | | 1.000 | |
|  | **Obstipation, hard/slow bowel movement** | 11 (22) | 17 (8) | | **0.008** | |
|  | **Impotence** | 3 (6) | 28 (13) | | 0.223 | |
|  | **Loss of sense of smell or taste** | 2 (4) | 18 (8) | | 0.385 | |
|  | **Chronic itching (for example due to dry skin)** | 5 (10) | 24 (11) | | 1.000 | |
|  | **Bedsores** | 1 (2) | 2 (1) | | 0.465 | |
|  | **Extreme/chronic fatigue** | 16 (32) | 60 (28) | | 0.602 | |
|  | **(Chronic) pain** | 16 (32) | 74 (34) | | 0.869 | |
|  | **Other** | 1 (2) | 27 (12) | | **0.037** | |
|  | **None of these** | 2 (4) | 6 (3) | | 0.646 | |
| **Medications^a^** | |  |  | |  | |
|  | **Antidepressants** | 15 (43) | 52 (29) | | 0.117 | |
|  | **Tranquillizers** | 4 (11) | 13 (7) | | 0.491 | |
|  | **Sleeping pills** | 8 (23) | 36 (20) | | 0.819 | |
|  | **Pain killers** | 15 (43) | 76 (43) | | 1.000 | |
|  | **Blood thinners** | 9 (26) | 61 (34) | | 0.431 | |
|  | **Anti-hypertensives** | 15 (43) | 81 (46) | | 0.853 | |
|  | **Anti-inflammatories** | 5 (14) | 31 (17) | | 0.807 | |
|  | **Blood sugar medication** | 9 (26) | 26 (15) | | 0.132 | |
|  | **Thyroid medication** | 7 (20) | 21 (12) | | 0.183 | |
|  | **Lung medication** | 9 (26) | 26 (15) | | 0.132 | |
|  | **Prostate medication** | 1 (3) | 7 (4) | | 1.000 | |
|  | **Other** | 6 (17) | 37 (21) | | 0.818 | |
|  | **None of these medications** | 0 (0) | 2 (1) | | 1.000 | |

Results are presented as N (%).

Percentages add up to more than 100% because respondents could select multiple diseases, complaints, and medications.

Statistically significant results (p < 0.05) are in bold. All were determined by Fisher’s exact tests.

^a^ N=35 and N=178 because respondents who reported not using any medications were not asked to list their medications.
