# Supplementary material for: Lifelong versus not lifelong death wishes in older adults without severe illness: a cross-sectional survey
Source: BMC Geriatr. 2022 Nov 21;22:885. doi: 10.1186/s12877-022-03592-5 (PMC9680128; doi:10.1186/s12877-022-03592-5)
Supplement: Supplementary file 4 — Additional file 4: Table2. Aspects strengthening the wish to die or to live. [file 12877_2022_3592_MOESM4_ESM.docx]

Additional table 2. Aspects strengthening the wish to die or to live

|  | | **L-PDW**  **(N=50)** N (%) | **NL-PDW (N=217)**  N (%) | **P-value** |
| --- | --- | --- | --- | --- |
| **Aspects strengthening the wish to die** | |  |  |  |
|  | **Diseases** | 22 (44) | 108 (50) | 0.128 |
|  | **Physical or mental deterioration** | 23 (46) | 117 (54) | 0.340 |
|  | **Increasing problems walking or with other movements** | 18 (36) | 88 (41) | 0.504 |
|  | **Limitation of my freedom** | 21 (42) | 90 (42) | 0.854 |
|  | **Being dependent on others** | 17 (34) | 90 (42) | 0.546 |
|  | **Sense of having little to no influence on my life** | 32 (64) | 116 (54) | 0.387 |
|  | **Feeling like I am a burden to others** | 19 (38) | 86 (40) | 0.970 |
|  | **Not enough good social contacts** | 22 (44) | 97 (45) | 0.247 |
|  | **Loss of my loved ones (e.g., through death, divorce)** | 12 (24) | 109 (50) | **0.002** |
|  | **Tension or arguments with people in my immediate environment** | 15 (30) | 60 (28) | 0.918 |
|  | **Loneliness** | 25 (50) | 116 (54) | 0.202 |
|  | **Not enough social activities** | 21 (42) | 92 (42) | 0.709 |
|  | **Loss (or lack) of status** | 5 (10) | 29 (13) | 0.365 |
|  | **Loss (or lack) of self-respect** | 26 (52) | 73 (34) | 0.062 |
|  | **No (or limited) future perspective** | 32 (64) | 147 (68) | 0.614 |
|  | **Boredom** | 11 (22) | 53 (24) | 0.627 |
|  | **Worrying** | 30 (60) | 143 (66) | 0.623 |
|  | **Bad memories (e.g., of traumatic experiences)** | 17 (34) | 85 (39) | 0.139 |
|  | **Move that is disappointing/turns out badly** | 5 (10) | 18 (8) | 0.810 |
|  | **Financial problems** | 16 (32) | 54 (25) | 0.596 |
|  | **Time of year** | 12 (24) | 46 (21) | 0.427 |
|  | **Something else** | 13 (26) | 33 (15) | 0.174 |
| **Aspects strengthening the wish to live** | |  |  |  |
|  | **Independence** | 31 (62) | 144 (66) | 0.760 |
|  | **Sense of freedom** | 33 (66) | 118 (54) | 0.382 |
|  | **Good social contacts, friendship** | 21 (42) | 108 (50) | 0.478 |
|  | **Sense of being connected to other people** | 22 (44) | 99 (46) | 0.899 |
|  | **Comfortable living conditions (nice house, nice neighbourhood)** | 33 (66) | 146 (67) | 0.217 |
|  | **Meaningful social activities** | 21 (42) | 81 (37) | 0.852 |
|  | **Volunteer work** | 10 (20) | 56 (26) | 0.332 |
|  | **Social engagement** | 17 (34) | 72 (33) | 0.910 |
|  | **Taking care of others** | 26 (52) | 102 (47) | 0.690 |
|  | **Making a difference for others** | 31 (62) | 124 (57) | 0.788 |
|  | **Feeling that I mean as much to the people around me as they do to me** | 18 (36) | 91 (42) | 0.412 |
|  | **Feeling useful** | 26 (52) | 95 (44) | 0.539 |
|  | **Sense of self-worth** | 25 (50) | 100 (46) | 0.843 |
|  | **Sense of self-respect** | 22 (44) | 96 (44) | 1.000 |
|  | **Being respected and appreciated by others** | 23 (46) | 110 (51) | 0.829 |
|  | **Good care** | 15 (30) | 79 (36) | 0.673 |
|  | **Good memories (e.g., of the past)** | 14 (28) | 114 (53) | **0.005** |
|  | **Humour, fun** | 34 (68) | 115 (53) | 0.168 |
|  | **Playing or watching sports** | 10 (20) | 47 (22) | 0.719 |
|  | **Sense that I am a part of a community** | 13 (26) | 55 (25) | 0.781 |
|  | **Sense that I am part of a larger whole** | 15 (30) | 44 (20) | **0.029** |
|  | **My worldview** | 12 (24) | 53 (24) | 0.919 |
|  | **My faith in God** | 5 (10) | 36 (17) | 0.534 |
|  | **The time of year/the season** | 17 (34) | 64 (30) | 0.113 |
|  | **Peace** | 31 (62) | 136 (63) | 0.425 |
|  | **Something else** | 8 (16) | 25 (12) | 0.204 |

Results are presented as N (%).

Percentages add up to more than 100% because respondents could select multiple aspects.

Statistically significant results (p < 0.05) are in bold. All were determined by Fisher’s exact tests.
