# Supplementary material for: Lifelong versus not lifelong death wishes in older adults without severe illness: a cross-sectional survey
Source: BMC Geriatr. 2022 Nov 21;22:885. doi: 10.1186/s12877-022-03592-5 (PMC9680128; doi:10.1186/s12877-022-03592-5)
Supplement: Supplementary file 5 — Additional file 5: Table3. Perspective on life. [file 12877_2022_3592_MOESM5_ESM.docx]

Additional table 3. Perspective on life

|  | | **L-PDW**  **(N=50)** N (%) | **NL-PDW (N=217)**  N (%) | **P-value** |
| --- | --- | --- | --- | --- |
| **Looking back ^a^** | |  |  |  |
|  | **I have had a good life** | 4 (3-6) | 5 (4-6) | **0.001** |
|  | **I have been mostly satisfied with my life** | 4 (2-6) | 5 (4-6) | **0.002** |
|  | **I have many good memories** | 4 (3-6) | 5 (4-7) | **0.000** |
|  | **I remember many negative experiences/events** | 5 (4-6) | 5 (4-6) | 0.762 |
| **Current situation ^b^** | |  |  |  |
|  | **Finding life worthwhile at this moment** |  |  |  |
|  | **Yes** | 29 (58) | 102 (47) | 0.209 |
|  | **No** | 21 (42) | 115 (53) |  |
|  | **I often feel lonely** | 4 (2-6) | 5 (3-6) | 0.154 |
|  | **People close to me need me** | 3 (2-5) | 4 (3-5) | 0.071 |
|  | **I am happy with my social contacts** | 4 (3-5) | 4 (3-5) | 0.837 |
|  | **I am attached to my pet(s)** | 6 (3-5) | 5 (2-7) | 0.068 |
|  | **I experience support from God/a higher power** | 1 (1-4) | 1 (1-4) | 0.971 |
|  | **There are plenty of things that make my life worthwhile** | 3 (2-5) | 4 (3-5) | 0.583 |
|  | **I contribute to society** | 4 (2-5) | 4 (2-5) | 0.455 |
|  | **As I grow older, I am appreciated less** | 4 (3-6) | 4 (3-6) | 0.501 |
|  | **I have sufficient opportunities to develop myself** | 4 (3-6) | 4 (3-5) | 0.120 |
|  | **I am able to do what suits me** | 4 (3-6) | 4 (3-6) | 0.913 |
|  | **I enjoy the everyday things** | 4 (3-5) | 4 (3-5) | 0.482 |
|  | **I am often bored** | 2 (1-4) | 3 (2-5) | 0.371 |
|  | **The burden of life weighs me down** | 5 (3-6) | 5 (4-6) | 0.387 |
|  | **I am afraid I will forget more and more** | 4 (2-6) | 4 (2-6) | 0.259 |
|  | **I regret things I did or neglected to do** | 4 (2-6) | 4 (3-6) | 0.155 |
|  | **I can take care of myself** | 6 (5-7) | 5 (4-6) | **0.016** |
|  | **I am becoming increasingly dependent on others** | 2 (1-5) | 4 (2-5) | **0.013** |
|  | **I have increasing doubts about my intellectual abilities** | 2 (1-4) | 2 (1-4) | 0.194 |
|  | **Care provided by others is an invasion of my privacy** | 4 (2-6) | 4 (3-5) | 0.768 |
|  | **In my life many things have happened that I had no say in** | 5 (4-7) | 5 (4-6) | 0.788 |
|  | **As I grow older, I feel I can be myself more and more** | 4 (3-6) | 4 (3-5) | 0.305 |
| **Looking at the future ^b^** | |  |  |  |
|  | **I worry about my own future** | 4 (3-6) | 5 (3-6) | 0.053 |
|  | **I worry about my partner’s or (grand)children’s future** | 3 (1-6) | 5 (3-6) | **0.002** |
|  | **I am curious to see what the future holds for me** | 3 (1-5) | 3 (2-5) | 0.086 |
|  | **I would prefer not to have to experience the future** | 5 (3-6) | 5 (4-6) | **0.039** |
|  | **Things can only go downhill as I grow older** | 5 (4-6) | 6 (5-7) | 0.086 |
|  | **I live by the day and I don’t think about the future** | 5 (3-6) | 5 (4-6) | 0.727 |

Results are presented as Median (Q1-Q3) except for the variable “Finding life worthwhile at this moment”, which is presented as N (%).

Medians are reported with 25th-75th percentiles.

Statistically significant results (p < 0.05) are in bold. All were determined by Kruskal-Wallis tests, except for the variable “Current situation” (Fisher’s exact test).

Except for the variable “Finding life worthwhile at this moment” respondents gave answers about their perspectives on life on a 7-point Likert scale. For the variable “Looking back” this scale ranged from 1 (“Totally disagree”) to 7 (“Strongly agree”) and for the other variables from 1 (“Not at all”) to 7 (“Very strong”). Respondents also had the option to answer “I do not know” (this answer is left out of the presentation of Medians (Q1-Q3) and not included in the tests).
