# Supplementary material for: Lifelong versus not lifelong death wishes in older adults without severe illness: a cross-sectional survey
Source: BMC Geriatr. 2022 Nov 21;22:885. doi: 10.1186/s12877-022-03592-5 (PMC9680128; doi:10.1186/s12877-022-03592-5)
Supplement: Supplementary file 6 — Additional file 6: Table 4. Good memories. [file 12877_2022_3592_MOESM6_ESM.docx]

Additional table 4. Good memories

|  | **L-PDW**  **(N=17)^a^** N (%)**^1^** | **NL-PDW (N=154)^a^**  N (%)**^2^** | **P-value** | |
| --- | --- | --- | --- | --- |
| **Happy memories with loved ones (like a birth, wedding day, family Christmas)** | 9 (53) | 98 (64) | 0.434 |  |
| **Successful moments (like graduation, promotion, being decorated, retirement, an anniversary)** | 3 (18) | 18 (12) | 0.443 |  |
| **A happy marriage/a happy relationship** | 9 (53) | 77 (50) | 1.000 |  |
| **Important achievements in my work** | 1 (6) | 30 (19) | 0.316 |  |
| **Moments that I was of significance to others** | 7 (41) | 48 (31) | 0.420 |  |
| **Sport achievements** | 0 (0) | 8 (5) | 1.000 |  |
| **Great vacations or travels** | 9 (53) | 86 (56) | 1.000 |  |
| **Healing/recovery from severe illness** | 1 (6) | 23 (15) | 0.473 |  |
| **Other** | 3 (18) | 7 (5) | 0.063 |  |

Results are presented as N (%).

Percentages add up to more than 100% because respondents could select multiple good memories.

Statistically significant results (p < 0.05) are in bold. All were determined by Fisher’s exact tests.

^a^ N=17 instead of 50 and N=154 instead of 217 respectively because only the respondents who had a score of 5 or higher on “I have many good memories” in table S3 were asked to list their good memories. Percentages in table S4 are based on N=17 and N=154.
