# Supplementary material for: Lifelong versus not lifelong death wishes in older adults without severe illness: a cross-sectional survey
Source: BMC Geriatr. 2022 Nov 21;22:885. doi: 10.1186/s12877-022-03592-5 (PMC9680128; doi:10.1186/s12877-022-03592-5)
Supplement: Supplementary file 7 — Additional file 7: Table5. Negative experiences or events. [file 12877_2022_3592_MOESM7_ESM.docx]

Additional table 5. Negative experiences or events

|  | **L-PDW**  **(N=30)^a^** N (%)**^1^** | **NL-PDW (N=138)^a^**  N (%)**^2^** | **P-value** |
| --- | --- | --- | --- |
| **Dismissal or unemployment** | 3 (10) | 11 (8) | 0.718 |
| **Failed career** | 4 (13) | 8 (6) | 0.230 |
| **Tensions or conflicts with my loved ones (e.g., spouse/partner, children, friends)** | 9 (30) | 38 (28) | 0.824 |
| **Broken contact with relative or friend** | 5 (17) | 23 (17) | 1.000 |
| **(Forced) move** | 0 (0) | 5 (4) | 0.587 |
| **Divorce** | 7 (23) | 25 (18) | 0.608 |
| **Illness** | 5 (17) | 39 (28) | 0.253 |
| **Childlessness** | 3 (10) | 3 (2) | 0.071 |
| **Death of a loved one** | 5 (17) | 42 (30) | 0.178 |
| **Attempted suicide of a loved one** | 0 (0) | 5 (4) | 0.587 |
| **Memories of war** | 0 (0) | 3 (2) | 1.000 |
| **Bad memories from my childhood** | 18 (60) | 57 (41) | 0.071 |
| **A trauma** | 10 (33) | 21 (15) | **0.035** |
| **Loss or bereavement** | 4 (13) | 50 (36) | **0.017** |
| **Financial problems** | 7 (23) | 31 (22) | 1.000 |
| **Other** | 3 (10) | 10 (7) | 0.705 |

Results are presented as N (%).

Percentages add up to more than 100% because respondents could select multiple negative experiences or events.

Statistically significant results (p < 0.05) are in bold. All were determined by Fisher’s exact tests.

^a^ N=30 instead of 50 and N=138 instead of 217 respectively because only the respondents who had a score of 5 or higher on “I remember many negative experiences/events” in table S3 were asked to list their negative experiences or events. Percentages in table S5 are based on N=30 and N=138.
