# Supplementary material for: Lifelong versus not lifelong death wishes in older adults without severe illness: a cross-sectional survey
Source: BMC Geriatr. 2022 Nov 21;22:885. doi: 10.1186/s12877-022-03592-5 (PMC9680128; doi:10.1186/s12877-022-03592-5)
Supplement: Supplementary file 8 — Additional file 8: Table 6. Life goals. [file 12877_2022_3592_MOESM8_ESM.docx]

Additional table 6. Life goals

|  | | **L-PDW**  **(N=50)** N (%) | **NL-PDW (N=217)**  N (%) | **P-value** |
| --- | --- | --- | --- | --- |
| **Importance of each life goal** | |  |  |  |
|  | **Live a healthy life** | 5 (4-6) | 5 (4-6) | 0.591 |
|  | **Be independent/self-reliant** | 6 (5-7) | 6 (5-7) | 0.816 |
|  | **Be able to decide how I live my life** | 7 (6-7) | 6 (6-7) | 0.491 |
|  | **Be involved in the community** | 5 (4-6) | 5 (4-6) | 0.848 |
|  | **Be of significance to others** | 5 (4-6) | 5 (4-6) | 0.206 |
|  | **Achieve something in life** | 4 (3-5) | 4 (3-5) | 0.827 |
|  | **Take care of others** | 5 (3-6) | 5 (4-6) | 0.137 |
|  | **Have/maintain friendships** | 5 (4-6) | 6 (4-6) | 0.211 |
|  | **Do what is right for me** | 6 (5-7) | 6 (5-7) | 0.311 |
|  | **Self development** | 5 (3-6) | 4 (3-6) | 0.463 |
|  | **Be appreciated, acknowledged** | 5 (4-6) | 5 (4-6) | 0.939 |
|  | **Enjoy life** | 5 (4-7) | 6 (4-7) | 0.641 |

Results are presented as Median (Q1-Q3).
Medians are reported with 25th-75th percentiles.

Statistically significant results (p < 0.05) are in bold. All were determined by Kruskal-Wallis tests.

7-point Likert scale ranging from 1 (“Very unimportant”) to 7 (“Very important”).
